# The Majority of Adult Pneumococcal Invasive Infections in Portugal Are Still Potentially Vaccine Preventable in Spite of Significant Declines of Serotypes 1 and 5

**DOI:** 10.1371/journal.pone.0073704

**Published:** 2013-09-16

**Authors:** Andreia N. Horácio, Jorge Diamantino-Miranda, Sandra I. Aguiar, Mário Ramirez, José Melo-Cristino, the Portuguese Group for the Study of Streptococcal Infections

**Affiliations:** Instituto de Microbiologia, Instituto de Medicina Molecular, Faculdade de Medicina, Universidade de Lisboa, Lisboa, Portugal; Rockefeller University, United States of America

## Abstract

In Portugal, pneumococcal conjugate vaccines have been administered to children outside of the national immunization plan since 2001. We determined the serotype and antimicrobial susceptibility of 1265 isolates responsible for adult invasive pneumococcal infections (IPD) between 2009 and 2011 and compared the results with previously published data from 1999 to 2008. Serotypes 3 (12.6%), 7F (10.0%), 19A (9.1%), 14 (8.4%), 1 (6.9%) and 8 (6.2%) were the most frequent and together accounted for 53.2% of adult IPD. Serotypes 1 and 5 declined significantly while serotype 34, not included in any vaccine, increased. Taken together, the serotypes included in the 13-valent conjugate vaccine (PCV13) peaked among adult IPD isolates in 2008 (70.2%) and declined since then reaching 53.5% in 2011. The decline in the serotypes included in the 23-valent polysaccharide vaccine since 2007 was also significant but much more modest with 79.2% of the isolates causing IPD in 2011 expressing these serotypes. Since the changes in serotypes causing IPD in adults coincided with the 10-valent and PCV13 introduction in children, it is unlikely that vaccination triggered these changes although it may have accelerated them. The proportion of IPD caused by serotypes included in the 7-valent conjugate vaccine remained stable (19.0%). Both penicillin non-susceptibility and erythromycin resistance increased in the study period, with serotypes 14 and 19A accounting for the majority of resistant isolates.

## Introduction

*Streptococcus pneumoniae* (pneumococcus) remains a significant cause of morbidity and mortality throughout the world affecting disproportionally the extremes of life. Prevention of these infections in persons ≥2 years belonging to risk groups and particularly among adults ≥65 years has relied on a vaccine including 23 of the 94 capsular polysaccharides known in pneumococci. Although older age is a recognized risk factor for pneumococcal disease, in Europe different countries have distinct recommendations regarding the use of the 23-valent polysaccharide vaccine (PPV23), ranging from the absence of national guidelines, to recommendations of universal or risk group vaccination starting at 60 or 65 years [1]. Perhaps because of an ongoing debate on PPV23 efficacy [2], [3], [4], in most European countries there is a low overall uptake of PPV23 [5]. In Portugal PPV23 uptake is at the lower end of the spectrum with estimates that approximately 10% of adults ≥65 years are vaccinated [6].

The remarkable efficacy of the seven-valent conjugate vaccine (PCV7) against the serotypes included in its formulation resulted in a sharp decline in the proportion of invasive pneumococcal infections (IPD) caused by these serotypes not only in vaccinated children [7], [8], [9], [10], [11], [12] but also across the entire population [6]. This “herd effect” is attributed to the reduced transmission of these serotypes from children to adults. In Europe, although there were epidemiological changes in the serotypes causing IPD in the non-vaccinated population in all countries where the vaccine was administered, the large reduction in the overall number of invasive infections in adults observed in the USA was not replicated in countries such as Spain, England and Wales and the Netherlands [10], [12], [13] where significant increases in non-vaccine serotypes (NVT) occurred.

Since the PCV7 serotypes represented a significant fraction of resistant isolates, vaccination was also anticipated to affect resistance. However, the effect of PCV7 on antibiotic resistance in Europe was variable. While a decrease in penicillin non-susceptibility was noted in all countries among isolates responsible for pediatric IPD in the post-PCV7 period [7], [13], [14], such decline was not apparent in adults in Portugal and Spain [6], [13], [14].

Serotype 19A has consistently been identified as a dominant non-vaccine serotype but other emerging non-vaccine serotypes differ between geographic locations and also between age groups [6], [7], [8], [9], [14]. Even within serotype 19A, different genetic lineages emerge in different geographic locations [15]. These data highlight the importance of the characteristics of the local pneumococcal population and of local selective forces in conditioning the outcomes of vaccination [16].

On the other hand, it is known that serotypes responsible for IPD may have significant temporal variations in the same geographic region as documented in Spain and Denmark [17], [18], even with limited antibiotic selective pressure and in the absence of PCV use. In addition, the divergent prevalence of the various serotypes in different geographic regions also conditions the potential benefits of vaccination. A much lower prevalence of serotype 1 IPD is documented in the USA than elsewhere [6], [9], [14], [18], [19], [20]. Although serotype 1 is frequently associated with outbreaks and significant yearly variations of the proportion of IPD caused by this serotype are documented, a considerable fraction of IPD was consistently caused by this serotype in the last decades in Europe [17], [18].

Two new pneumococcal conjugate vaccine (PCV) formulations are now commercially available and used in children. A 10-valent formulation (PCV10) including, in addition to the PCV7 serotypes, serotypes 1, 5 and 7F and a 13-valent conjugate vaccine (PCV13), including all PCV10 serotypes plus serotypes 3, 6A and 19A. The introduction of these vaccines into clinical practice has the potential to once again change the characteristics of pediatric IPD, with initial data showing the capacity of PCV13 to blunt or even reverse the rise of some of the most successful serotypes that have emerged as causes of pediatric IPD since the introduction of PCV7 [21], [22], [23].

PCV13 was recently licensed for use in adults ≥50 years and this was soon followed by a recommendation in the USA for its use in adults with immunocompromising conditions [24]. Approval of PCV13 for adults was based on immunogenicity studies and the results of a large study that is currently underway in the Netherlands [25] comparing it to placebo for the prevention of vaccine-serotype community acquired pneumonia in adults are expected to become available in late 2013. The observed benefits of conjugate vaccines in children launched a discussion about the potential benefits of vaccinating the adult population with these vaccines instead of PPV23 [2], [3], [4], [26]. Independently of the immunological arguments, the potential benefits of adult vaccination with either PCV13 or PPV23 are a moving target since secular trends in pneumococcal serotypes and the herd effect provided by PCV7, and now also hoped for the use of PCV13 in children, would be expected to reduce the importance of the serotypes included in conjugate vaccines in adult IPD.

In Portugal PCVs were not included in the National Immunization Plan but there has been a steady increase in PCV7 uptake since 2001, reaching 75% of children ≤2 yrs in 2008 [14]. The expanded valency PCVs for childhood vaccination – PCV10 and PCV13– became available in mid-2009 and early-2010, respectively. In previous studies, we showed that significant changes in the serotypes causing IPD in children followed PCV7 availability [7], [14] and that there was evidence for a herd effect in the adult population [6], [14]. This study aimed at documenting the continued changes on serotype distribution and antimicrobial resistance in different adult groups and evaluating the proportion of potentially vaccine preventable adult IPD in Portugal immediately prior to the approval in January 2012 of PCV13 use in adults >50 yrs.

## Materials and Methods

### Ethics Statement

Case reporting and isolate collection were considered to be surveillance activities and were exempt from evaluation by the Review Board of the Faculdade de Medicina da Universidade de Lisboa.

### Bacterial Isolates

Since 1999, the Portuguese Group for the Study of Streptococcal Infections has monitored pneumococci causing invasive infections in Portugal. This is a laboratory-based surveillance system, in which 30 microbiology laboratories throughout Portugal are asked to identify all isolates responsible for IPD and to send them to a central laboratory for characterization. A case of invasive disease is defined by an isolate of *S. pneumoniae* recovered from a normally sterile body site such as blood or CSF. Although the laboratories were contacted periodically to submit the isolates to the central laboratory, no audit was performed to ensure compliance, which may be variable in this type of study. Isolates recovered up to 2008 were previously characterized [6], [14], [27]. Only isolates recovered from adult invasive infections, i.e. recovered from patients ≥18 yrs, between 2009 and 2011 were included in the present study. One isolate from each patient in each year was considered. All strains were identified as *S. pneumoniae* by colony morphology and hemolysis on blood agar plates, optochin susceptibility and bile solubility.

### Serotyping and Antimicrobial Susceptibility Testing

Serotyping was performed by the standard capsular reaction test using the chessboard system and specific sera (Statens Serum Institut, Copenhagen, Denmark). Serotypes were grouped into conjugate vaccine serotypes, i.e., those included in PCV13 (serotypes 1, 3, 4, 5, 6A, 6B, 7F, 9V, 14, 18C, 19F, 19A, 23F) and that comprise all the serotypes found in the lower valency vaccines, those included in PPV23 (all serotypes included in PCV13 except 6A and serotypes 2, 8, 9N, 10A, 11A, 12F, 15B, 17F, 20, 22F and 33F), and non-vaccine serotypes (NVT). Etest strips (AB Biodisk, Solna, Sweden) were used to determine the MICs for penicillin and cefotaxime. In 2008, the CLSI changed the recommended breakpoints used to interpret MIC values. Unless otherwise stated we have used the CLSI-recommended breakpoints prior to 2008 [28] as epidemiological breakpoints that allow the comparison with previous studies. According to these recommendations, intermediate level penicillin resistance was defined as MIC 0.12–1.0 µg/ml and high level resistance as MIC ≥2.0 µg/ml. Isolates that fell into either of these classes were designated penicillin non-susceptible. Susceptibility to cefotaxime was defined as MIC ≤1.0 µg/ml for non-meningitis cases and an MIC ≤0.5 µg/ml for meningitis cases.

Isolates were further characterized by determining their susceptibility to erythromycin, clindamycin, vancomycin, linezolid, tetracycline, levofloxacin, trimethroprim-sulfamethoxazole and chloramphenicol by the Kirby-Bauer disk diffusion technique, according to the CLSI recommendations and interpretative criteria [29].

Macrolide resistance phenotypes were identified using a double disc test with erythromycin and clindamycin according to a previously published procedure [30]. Simultaneous resistance to erythromycin and clindamycin defines the MLS_B_ phenotype (resistance to macrolides, lincosamides and streptogramin B) while non-susceptibility only to erythromycin indicates the M phenotype.

The prevalence of the various serotypes was compared with already published data from 1999–2008 [6], [14], [27]. We established previously that no significant changes in serotype distribution occurred until 2003 in adult IPD and have therefore considered 1999–2003 as the pre-vaccine period [14].

### Statistical Analysis

Simpson’s index of diversity (SID) and respective 95% confidence intervals (CI_95%_) was used to measure the population diversity [31]. Adjusted Wallace (AW) coefficients were used to compare two sets of partitions [32]. The calculation of these indices was done using the online tool www.comparingpartitions.info. Differences were evaluated by the Fisher exact test and the Cochran-Armitage test was used for trends with the false discovery rate (FDR) correction for multiple testing [33]. A p<0.05 was considered significant for all tests.

## Results

### Isolate Collection

Between 2009 and 2011 a total of 1265 isolates were recovered from normally sterile sites: 448 in 2009, 404 in 2010 and 413 in 2011. Isolates were recovered from blood (n = 1121, 88.6%), CSF (n = 97, 7.7%), pleural fluid (n = 30, 2.4%), peritoneal fluid (n = 10, 0.8%) and other normally sterile sites (n = 7, 0.5%). Regarding age distribution, 353 isolates (27.9%) were recovered from patients 18–49 yrs, 272 (21.5%) from patients 50–64 yrs and 640 (50.6%) from patients ≥65 yrs.

The 1265 isolates recovered in 2009–2011 are in line with the 1100 isolates recovered in 2006–2008 and reported previously [6]. This suggests that the surveillance network is stable and that no major changes are affecting IPD reporting in the two periods. However, although unlikely, we cannot completely exclude the possibility that there was an increase in reporting that may have compensated for a potential decrease in IPD incidence.

### Serotype Distribution

We detected 50 different capsular types among the 1265 isolates. The most frequent, that accounted for 53.2% of all adult IPD, were serotypes 3 (n = 160, 12.6%), 7F (n = 126, 10.0%), 19A (n = 115, 9.1%), 14 (n = 106, 8.4%), 1 (n = 87, 6.9%) and 8 (n = 79, 6.2%). During the study period (2009–2011), the only significant changes found in individual serotype prevalence after FDR correction were of serotype 1, that decreased from 10.7% to 4.1% (Cochran-Armitage test of trend *p*<0.001), serotype 5, that decreased from 2.0% to 0% (Cochran-Armitage test of trend *p* = 0.003) and serotype 34, that did not cause any invasive infections in 2009 and 2010 but was detected in 1.9% of the isolates causing IPD in 2011 (Cochran-Armitage test of trend *p*<0.001).

**Figure 1:**
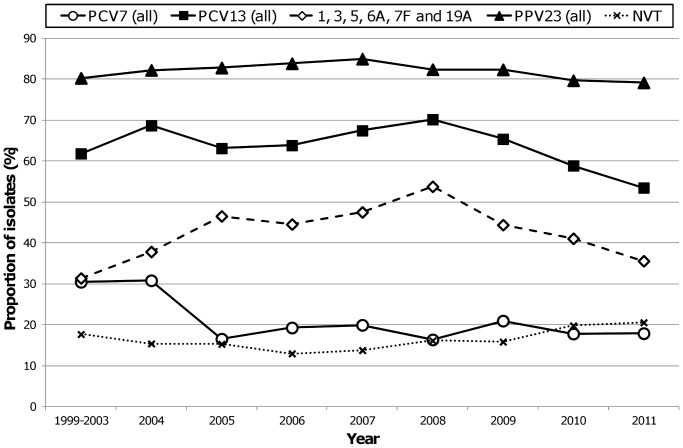
Proportion of isolates expressing serotypes included in pneumococcal vaccines causing invasive infections in adults in Portugal (1999–2011). The data up to 2008 were presented previously [6], [14], [27]. The period of 1999–2003, previously identified as the pre-PCV7 period [14], was analyzed together.

Fig. 1 shows the evolution of vaccine preventable IPD between 1999 and 2011. For the period 1999–2003, defined previously as the pre-PCV7 period [14], the results were averaged over the entire period. After the significant decline of IPD caused by PCV7 serotypes between 2004 and 2005, from 30.8% to 16.5% (*p*<0.001), a steady and low prevalence was seen until 2011. As previously documented [6], in spite of the decrease of PCV7 serotypes, the increase in serotypes 1, 19A and 7F resulted in an overall increase in PCV13 serotypes in the post-PCV7 period, from 61.9% in 1999–2003 to 70.2% in 2008 (Cochran-Armitage test of trend *p* = 0.014). However, 2008 was an inflection point (Fig. 1) and the proportion of isolates presenting PCV13 serotypes started to decline from then onwards such that in 2011 only 53.5% of the isolates presented PCV13 serotypes (from 2008 to 2011, Cochran-Armitage test of trend *p*<0.001). This change was mainly driven by a decrease in prevalence of serotypes 1 and 5 from 13.5% and 2.9% in 2008 to 4.1% and 0% in 2011, respectively (Cochran-Armitage test of trend *p*<0.001 for both, significant after FDR) (Table S1).

The proportion of PPV23 serotypes also increased slightly but non-significantly up to 2007. From 2007 onwards there was a slight but significant decrease in the proportion of IPD caused by PPV23 serotypes, from 85.0% in 2007 to 79.2% in 2011 (Cochran-Armitage test of trend *p* = 0.018). Initially this decline occurred in spite of the increase in PCV13 serotypes that peaked in 2008. Later, the decline of PCV13 serotypes was opposed by an important increase in the proportion of IPD caused by the additional serotypes found in PPV23 but absent from PCV13, from 13.7% in 2008 to 25.9% in 2011 (Cochran-Armitage test of trend *p*<0.001). When looking individually at these serotypes, although several increased in frequency, only serotype 8 increased significantly from 3.7% in 2008 to 8.0% in 2011 (Cochran-Armitage test of trend *p*<0.002, significant after FDR).

**Figure 2:**
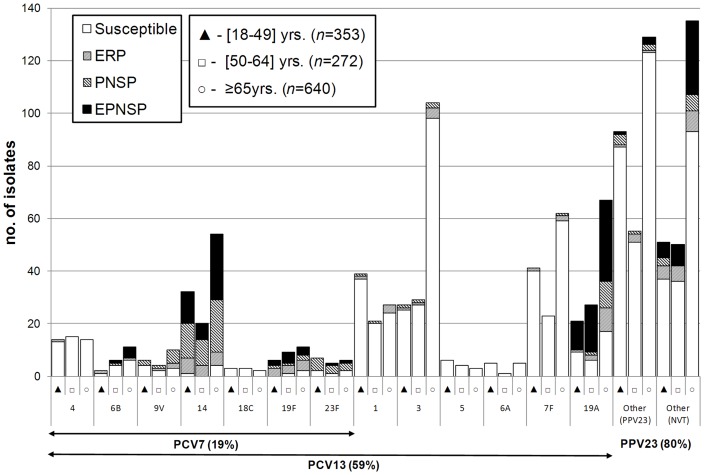
Number of isolates expressing serotypes included in conjugate vaccines causing invasive infections in Portugal (2009–2011). The number of isolates expressing each serotype in each of the age groups considered is indicated. Isolates recovered from patients 18 to 49≥65 yrs are indicated by open circles. Isolates presenting both erythromycin resistance and penicillin non-susceptibility (EPNSP) are represented by closed black bars. Penicillin non-susceptible isolates (PNSP) are indicated by dark hatched bars. Erythromycin resistant pneumococci(ERP) are indicated by light hatched bars. Isolates susceptible to both penicillin and erythromycin are represented by white open bars. The serotypes included in each of the conjugate vaccines are indicated by the arrows. NVT – non-vaccine serotypes, i.e., serotypes not included in any of the currently available vaccines (PCV13 and PPV23). Twenty-eight NVT were detected representing 236 isolates as follows: 6C (n = 36); 23A (n = 20); 12B (n = 19); 16F (n = 18); 23B and 33A (n = 16 each); 15A and 29 (n = 15 each); 24F (n = 13); non-typable (n = 10); 31, 34 and 35F (n = 8 each); 7C, 25A and 35B (n = 5 each); 21 (n = 4); 18A (n = 3); 13 and 17A (n = 2 each); 7A, 11C, 15F, 24A, 25F, 28A, 28F and 37 (n = 1 each).

**Figure 3:**
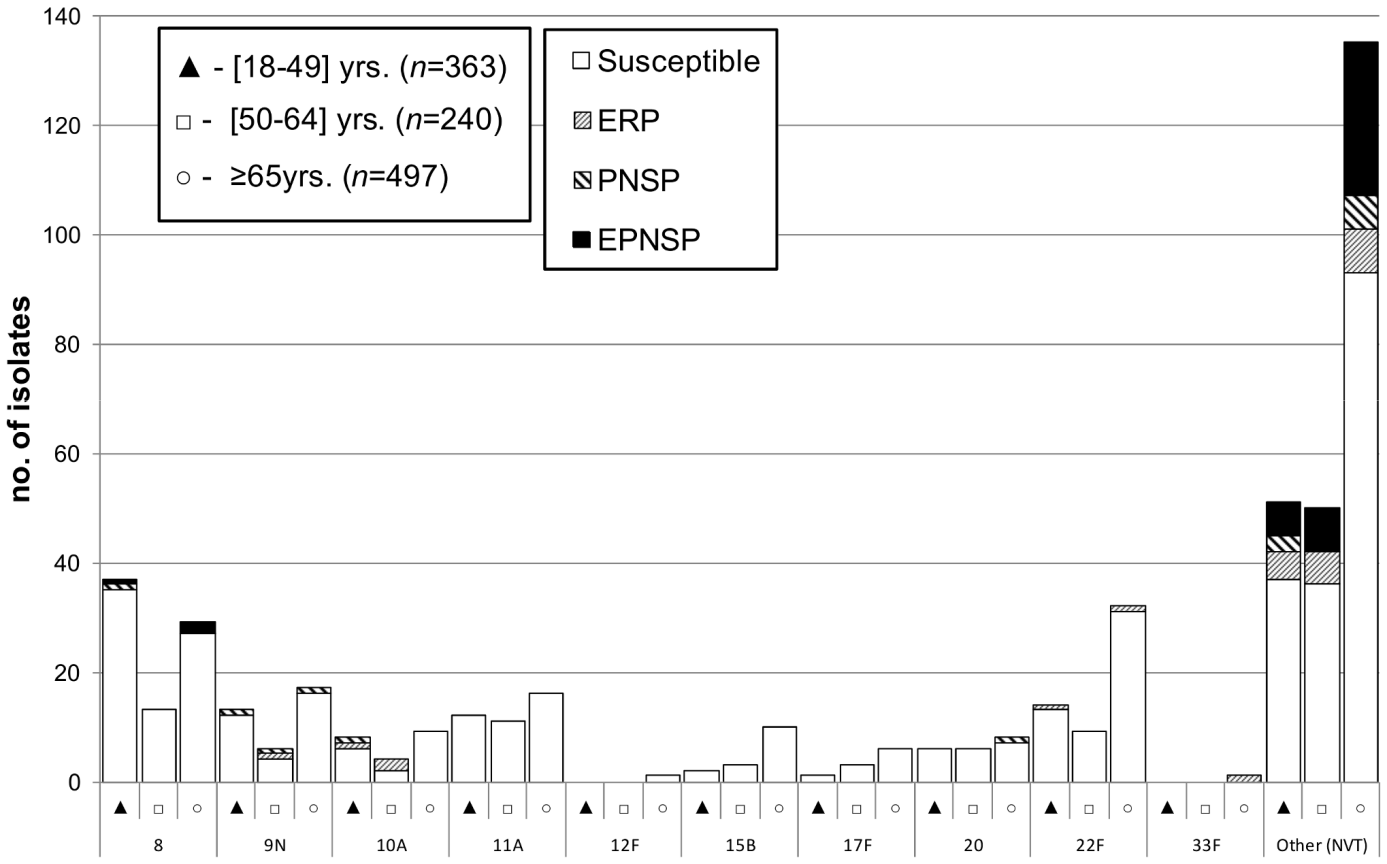
Number of isolates expressing serotypes present in the 23-valent polysaccharide vaccine but not included in conjugate vaccines causing invasive infections in Portugal (2009–2011). See the legend of figure 1. Out of the 11 serotypes present in the 23-valent polysaccharide vaccine PPV23 but absent from the 13-valent conjugate vaccine PCV13, serotype 2 was not found in our collection.

Fig. 2 shows the distribution of the individual serotypes included in the conjugate vaccines, stratified by the age group of the patients. Fig. 3 shows the distribution of the additional serotypes found in PPV23 that are not included in the conjugate vaccines. To analyze the serotype diversity within each age group, SIDs were calculated. The serotypes of the isolates causing invasive infections in any of the age groups considered were highly diverse (18–49 yrs [SID: 0.939, CI_95%_: 0.930–0.947]; 50–64 yrs [SID: 0.949, CI_95%_: 0.941–0.958]; ≥65 yrs [SID: 0.934, CI_95%_: 0.926–0.943]). The only significant difference was a higher diversity of serotypes in the 50–64 yrs age group relative to ≥65 yrs age group (*p* = 0.013). A similar analysis was performed for determining the serotype diversity in each study year but no significant changes occurred between 2009 and 2011 nor were changes in diversity noted between the 2004–2008 period and 2009–2011 (data not shown).

Although the frequency of each serotype varies according to the age groups considered, only for serotypes 1, 3, 8 and 19A were these differences significant. While the frequency of serotype 1 decreases with age (18–49 yrs –11.0%, 50–64 yrs –7.7%, ≥65 yrs –4.2%; Cochran-Armitage test of trend *p*<0.001), the frequency of serotype 3 increases with age (18–49 yrs –7.6%, 50–64 yrs –10.7%, ≥65 yrs –16.3%; Cochran-Armitage test of trend *p*<0.001). For serotypes 8 and 19A a trend with age was not evident. However, serotype 8 was more frequent in the youngest group (18–49 yrs –10.5%) than in either of the oldest age groups (50–64 yrs –4.8%, *p* = 0.011 and ≥65–4.5%, *p*<0.001), and serotype 19A showed a higher prevalence in older adults (≥65 yrs; 10.5%) than in younger adults (18–49 yrs; 5.9%, *p* = 0.019). Taking together the three years of the study (2009–2011), the proportion of IPD caused by the serotypes included in both conjugate vaccines and in PPV23 was roughly the same in all age groups considered (Table 1).

**Table 1:** Isolates expressing serotypes included in pneumococcal vaccines (2009–2011).

		No. isolates (%)		
		18–49 yrs	50–64 yrs	≥65 yrs
	2009	24 (18.2)	21 (22.8)	49 (21.9)
PCV7	2010	22 (19.6)	20 (24.7)	30 (14.2)
	2011	24 (22.0)	21 (21.2)	29 (14.1)
	2009	80 (60.6)	63 (68.5)	150 (67.0)
PCV13	2010	65 (58.0)	48 (59.3)	125 (59.2)
	2011	64 (58.7)	56 (56.6)	101 (49.3)
	2009	104 (78.8)	78 (84.8)	187 (83.5)
PPV23	2010	97 (86.6)	66 (81.5)	159 (75.4)
	2011	96 (88.1)	77 (77.8)	154 (75.1)

When analyzing individual serotypes with three or more CSF isolates, a positive association with CSF was found for serotypes 6B (*p* = 0.002), 16F (*p* = 0.009) and 19F (*p* = 0.002), all significant after FDR (Table S2).

### Antimicrobial Susceptibility

Resistance to the tested antimicrobials is summarized in table 2 and figures 2 and 3. Overall 266 isolates (21.0%) were penicillin non-susceptible pneumococci (PNSP) –205 (16.2%) expressed low level resistance (MIC = 0.12–1.0) and 61 (4.8%) high level resistance (MIC ≥2 µg/ml). Considering current CLSI breakpoints for parenteral penicillin where susceptibility is defined as MIC <0.06 µg/ml for meningitis cases [29], 17 isolates (17.5%) from CSF would have been considered resistant and 27 isolates (2.3%) from non-meningitis cases would have been considered non-susceptible –24 (2.1%) intermediately resistant and 3 (0.3%) fully resistant. Erythromycin resistant pneumococci (ERP) accounted for19.4% of the isolates (n = 245), of which 194 isolates (79.2%) expressed the MLS_B_ phenotype and 51 (20.8%) the M phenotype. All isolates were susceptible to vancomycin and linezolid. The simultaneous expression of erythromycin resistance and penicillin non-susceptibility (EPNSP) was found in 13.0% of the isolates.

**Table 2:** Antimicrobial resistance of the isolates responsible for invasive infections in adults (2009–2011).

	No. resistant isolates (%)^a^		
	18–49 yrs	50–64 yrs	≥65 yrs
	(n = 353)	(n = 272)	(n = 640)
PEN	62 (17.6)	58 (21.3)	146 (22.8)
MIC_90_	1	1	1
MIC_50_	0.023	0.023	0.023
CTX	14 (4.0)	12 (4.4)	24 (3.8)
MIC_90_	0.75	0.75	0.75
MIC_50_	0.023	0.023	0.023
LEV	0 (0)	0 (0)	2 (0.3)
ERY	53 (15.0)	58 (21.3)	134 (20.9)
CLI	40 (11.3)	51 (18.8)	108 (16.9)
CHL	7 (2.0)	11 (4.0)	11 (1.7)
SXT	68 (19.3)	62 (22.8)	117 (18.3)
TET	49 (13.9)	51 (18.8)	118 (18.4)

a PEN – penicillin; CTX – cefotaxime; LEV – levofloxacin; ERY – erythromycin; CLI – clindamycin; CHL – chloramphenicol; SXT – trimethoprim/sulphamethoxazole; TET – tetracycline. All isolates were susceptible to vancomycin and linezolid.

Resistance to penicillin, erythromycin and clindamycin increased with the age group considered (table 2). While the increase in erythromycin and clindamycin were statistically supported (Cochram-Armitage test of trend, *p* = 0.035 and *p* = 0.039, respectively) the increase in penicillin non-susceptibility was not (*p* = 0.057). For the other tested antimicrobials no significant differences were noted. The proportions of PNSP and ERP in 2009–2011 were higher than those previously reported (Figure S1). The proportion of PNSP increased from being previously stable (from an average value of 16.7% in 1999–2008 to 21.0% in 2009–2011, *p* = 0.002). On the other hand, there was a consistent increase in the proportion of ERP since PCV7 introduction (from 1999–2003 to 2011, Cochran-Armitage test of trend *p*<0.001). Although the overall proportion of ERP has been increasing, when analyzing changes within the study period a significant decrease was noted from 2010 to 2011, from 23.8% to 15.7% (*p* = 0.005). Similarly, the proportion of PNSP also decreased from 23.8% in 2010 to 19.6% in 2011, but this change was not statistically significant (*p* = 0.174).

The correlation between serotype and antimicrobial resistance was high. The AW for serotype and penicillin non-susceptibility was 0.539 (CI_95%_ 0.476–0.601), higher than the AW for serotype and erythromycin resistance (0.403, CI_95%_ 0.336–0.470). Together, serotypes 19A and 14 contributed greatly to penicillin non-susceptibility (59.0%) and to erythromycin resistance (53.1%) (Fig. 2). Serotypes included in PCV7 represented 47.4%, 36.7% and 35.8% of PNSP, ERP and EPNSP, respectively, while serotypes included in PCV13 constituted 76.7%, 71.4% and 72.1%, respectively. Considering the serotypes included in PPV23, only a modest increase relative to the PCV13 serotypes is noted (80.2%, 75.1% and 74.5%, of PNSP, ERP and EPNSP, respectively) since most of the remaining resistant isolates express NVTs (Fig. 3). Among the EPNSP expressing NVTs, serotypes 6C (45.2%) and 15A (28.6%) were dominant.

## Discussion

The effect of childhood vaccination in the distribution of IPD serotypes in adults was always found to be delayed in relation to the effects seen in children in the countries where the vaccine is available [12], [14], [34]. Given this prior experience with PCV7, the introduction of PCV13 in childhood vaccination in early 2010 in Portugal would only be expected to have an effect in the distribution of serotypes causing IPD in adults around 2011. If one considers the serotypes common to PCV10, then the introduction of PCV10 in childhood vaccination in mid-2009 would raise the possibility that an effect on these serotypes could occur earlier. However, the significant increase in adult IPD in the post-PCV7 period of serotypes 1, 7F (common to both PCVs) and 19A (exclusively found in PCV13) peaked in 2008 [6]. While serotypes 7F and 19A remained stable from then onwards, the number of serotype 1 isolates started to decline in 2009 (Table S1). This was accompanied by a significant reduction of serotype 5 and a strong reduction of serotype 6A (not included in PCV10) in the same period, that were responsible for the overall fall in PCV13 serotypes (Fig. 1). The fact that the decline started in 2009, before PCV13 introduction and shortly after PCV10 became available, strongly argues that the changes seen here were not triggered by the use of PCVs, although they may have been accelerated by PCV use.

Serotype 3 remains the most prevalent serotype in adult IPD after PCV7 introduction and continued to be significantly associated with older adults [6]. Similarly, serotype 7F remains the second most frequently identified serotype. Although serotypes 3 and 7F were not as frequent, these were also important among IPD in children [7] and were commonly found among patients with pleural parapneumonic effusion of all ages in the same period (unpublished data), attesting to their virulence. In spite of their continued dominance, both serotype 3 and 7F isolates remain mostly susceptible to all tested antimicrobials (Fig. 1), as previously described in Portugal and elsewhere [18], [35].

The increase in serotype 19A as a cause of IPD in both children [7] and adults [6] plateaued in the study period in adults with the overall proportion of IPD isolates expressing this serotype remaining stable at around 9%. Serotype 19A as a whole, did not have an enhanced propensity to cause invasive infections [36], but particular lineages within this serotype were found to have different preferences in their association with the human host [15]. In Portugal it was shown that the lineage that was expanding was associated with antimicrobial resistance [15], as was also seen here (Fig. 2). While no association with particular adult age groups was seen previously for serotype 19A IPD, this serotype was now significantly associated with older adults (≥65 yrs) rather than younger adults (18–49 yrs). This could be driven in part by a higher antimicrobial consumption in this age group, a trend that may be emerging in developed countries [37]. However, in Norway the post-PCV7 rise of serotype 19A was mostly dominated by a penicillin susceptible clone [38], suggesting that selection for antimicrobial resistance alone cannot explain the post-PCV7 rise of this serotype.

Despite 10 years of PCV7 use in children, serotype 14 was still responsible for a significant fraction of IPD in all adult age groups (Fig. 2) in contrast to what happens elsewhere, where more significant reductions of serotype 14 in adult IPD followed PCV7 use in children [12], [34]. Serotype 14 isolates were mostly resistant to either erythromycin or penicillin or both (101/106, 95%) (Fig. 2). A high proportion of penicillin non-susceptibility, erythromycin resistance and erythromycin and penicillin non-susceptibility has been a characteristic of serotype 14 isolates since before PCV7 introduction [14], [27], [35], a feature that was accentuated in the post-PCV7 period in both pediatric and adult IPD [6], [7].

Serotype 1 traditionally accounts for a higher proportion of pediatric IPD in Europe than in North America. In Portugal serotype 1 was the second most important serotype in adult IPD between 2006–2008 [6]. In neighboring Spain, outside the Madrid area where PCV7 was available but not included in the national immunization plan similar to Portugal, an increased importance of serotype 1 in IPD both in the group targeted for PCV7 vaccination as well as in older children and adults was also documented [39]. The decline in the importance of serotype 1 as a cause of IPD with age in adults reported previously [6] was also seen in this period. However, serotype 1 dropped from the second most frequent overall cause of IPD in adults to fifth. As discussed above, the trigger of this decline cannot be solely attributed to a possible herd effect of PCV use in children since it started before vaccination of children with the expanded valency PCVs that include this serotype. Since serotype 1 remains mostly susceptible to antimicrobials (Fig. 2), the continued pressure of antimicrobial use could be invoked to explain this reduction. However, this does not seem plausible since serotypes 3, 4 and 7F, all also included in PCV13 and mostly susceptible to antimicrobials similarly to serotype 1 isolates, remain important and stable serotypes in IPD in adults in Portugal in the post-PCV7 period. Serotypes 1 and 7F were found to have an enhanced invasive disease potential [36] and were thus candidates to increase in prevalence in IPD in the post-PCV7 period, as indeed happened [6]. The decline in serotype 1 could be due to unexplained temporal trends that have been known to affect this serotype [17], [18]. These temporal trends may have subsequently been accelerated by PCV use.

Two other serotypes with enhanced invasive disease potential – serotypes 5 and 8 [36]– showed opposite trends. Serotype 5 was shown to cause outbreaks in open communities [40] and a significant increase in cases had been noted in 2008, although we have no evidence that these cases correspond to an outbreak. The subsequent decline of serotype 5 isolates could then be the natural dynamics of a putative outbreak. The suggestion that PCV use could have contributed to the decline of serotype 5 is supported by the observation that 2011 is the only year since surveillance started in 1999 when no isolates expressing serotype 5 were detected among IPD cases in adults. Serotype 8 on the other hand is a non-PCV serotype that has been increasing since 2008 and that is now the sixth most frequent serotype in IPD in adults (Fig. 3). Serotype 34 is a NVT that increased in the study period, although it is responsible for a modest number of cases. Although this serotype as a whole was associated with carriage, different lineages expressing this serotype showed distinct capacities to cause invasive disease [36]. It is therefore possible that its increase in IPD documented here is driven by a limited expansion of particularly virulent lineages. The other non-PCV serotypes that are among the ten most frequently found in adult IPD (Fig. 3) have all increased in frequency, although this was not statistically supported, and included the PPV23 serotypes 22F, 11A and 9N and the NVT serotype 6C. The latter was also notable for being frequently resistant to both erythromycin and penicillin. Serotypes 6C and 22F were also found among the most frequent in adult IPD in Canada in 2010 [34]. Since these are not covered by currently available PCVs, these serotypes may emerge as important causes of adult IPD in the post-PCV era.

In spite of the large reduction in the number of PCV7 serotypes, the five serotypes included in this vaccine and that were traditionally associated with resistance (6B, 9V, 14, 19F and 23F), still accounted for a significant fraction of isolates resistant to either erythromycin, penicillin or both (45%), and this proportion declined only slightly from what was seen in 2006–2008 (47%) [6]. Both penicillin and erythromycin non-susceptibility, that is concentrated in the serotypes included in PCVs, has risen in adult IPD. The emergence of multiresistant serotype 19A isolates in the post-PCV7 period in adult IPD played a major role in preventing the decline of resistance in IPD in Portugal by compensating the decline of resistant isolates expressing PCV7 serotypes. The significant decrease of erythromycin resistance noted in 2011 may signal a change in this trend, but there were multiple serotypes responsible for this decline and no significant concentration in PCV13 serotypes was noted.

In contrast to our expectations, the use of PCV7 and now of PCV13 has not resulted in further declines of the PCV7 serotypes as causes of adult IPD. The continued importance of these serotypes is in contrast to the massive declines that lead to the almost elimination of PCV7 serotypes as causes of adult IPD in the USA [9], [16]. The reasons behind this difference are possibly multifactorial and may include: 1) differences in the transmission dynamics of these serotypes in the USA and Portugal; 2) differences in the clonal composition or in the selective pressures exerted upon the pneumococcal populations; 3) a relatively slow uptake of PCV7 in Portugal when compared to the USA; and 4) a lower coverage of PCV7 vaccination in children in Portugal. Although we cannot formally exclude the first two possibilities, we believe that their impact would be transient since the pneumococcal population would adapt to these new circumstances. On the other hand, the later two possibilities are particularly interesting and suggest that in order to obtain the full public health benefits of vaccinating children with PCV13, one should aim at the rapid introduction of the vaccine and at vaccination coverage higher than the 75% currently achieved in Portugal. The proportion of PCV7 serotypes has shown little change since 2005, when the proportion of PCV7 serotypes causing IPD in adults declined from pre-PCV7 levels to its current values. It is therefore likely that the stability of PCV7 serotypes in the intervening six years corresponds to a new steady-state related to the lower vaccination coverage of children in Portugal relative to that in the USA or England and Wales [12], [19].

The recent replacement with the 13-valent formulation for the vaccination of children in Portugal, as has happened in many countries, may equally lead to herd effects in the additional serotypes included in this vaccine. The approval for the use of PCV13 in adults raises the possibility that adult vaccination may not only reduce IPD due to vaccine serotypes in vaccinees but also lead to reduced carriage of the serotypes included in the vaccine, as was seen in children, further compounding the herd effect noted from childhood vaccination. However, even without adult vaccination, continued use of conjugate vaccines in children and the herd effects they produce together with sustained high antimicrobial usage, are likely to drive alterations in the serotypes causing adult IPD that will influence the fraction of potentially vaccine preventable disease in this age group.

Our study was not designed to allow the estimate of the incidence of IPD and it therefore does not evaluate potential changes in incidence with time and in particular since PCV7 introduction. However, the design based on the reporting of all laboratory confirmed IPD cases with isolation of the etiological agent within the surveillance network, the large number of isolates studied, the wide coverage of the country by the network and the stable number of isolates reported in each year, guarantees that the data accurately represents IPD in Portugal and can be used to evaluate changes in the relative importance of the different serotypes. PPV23 availability since 1996 had only minor effects on the proportion of adult IPD caused by PPV23 serotypes but due to its limited use in the country [6] maybe none should be expected. The proportion of infections potentially covered by PPV23 in Portugal remained always above 80% since surveillance was started in 1999, except in 2011 when it dropped slightly below that level (Fig. 1). This occurred despite significant serotype changes in this period. In contrast, the fraction of adult IPD potentially preventable by PCV13 that had increased diminished in recent years reaching 53.5% in 2011 and may be even lower in patients with co-morbidities [41]. PCV13 use could still potentially prevent more than half of adult IPD in Portugal at the time it was licensed for use in adults >50 yrs. The dynamics of the serotypes causing IPD in adults justify the continued surveillance of these infections in order to evaluate the potential coverage afforded by each of the two currently available vaccines with an adult indication.

## Supporting Information

Figure S1 **Proportion of penicillin non-susceptible pneumococci (PNSP) and erythromycin resistant pneumococci (ERP) (1999–2011).** The period 1999–2003, previously identified as the pre-PCV7 period, was analyzed together.
(PDF)

Table S1 **Number of isolates expressing serotypes included in the 13-valent conjugate vaccine but not included in the 7-valent conjugate vaccine causing invasive infections in Portugal (2008–2011).**
(PDF)

Table S2 **Capsular types of the isolates recovered from CSF between 2009 and 2011.**
(PDF)
