# Supplementary material for: The Majority of Adult Pneumococcal Invasive Infections in Portugal Are Still Potentially Vaccine Preventable in Spite of Significant Declines of Serotypes 1 and 5
Source: PLoS One. 2013 Sep 16;8(9):e73704. doi: 10.1371/journal.pone.0073704 (PMC3774749; doi:10.1371/journal.pone.0073704)
Supplement: Figure S1 — Proportion of penicillin non-susceptible pneumococci (PNSP) and erythromycin resistant pneumococci (ERP) (1999–2011). The period 1999–2003, previously identified as the pre-PCV7 period, was analyzed together. (PDF) [file pone.0073704.s001.pdf]

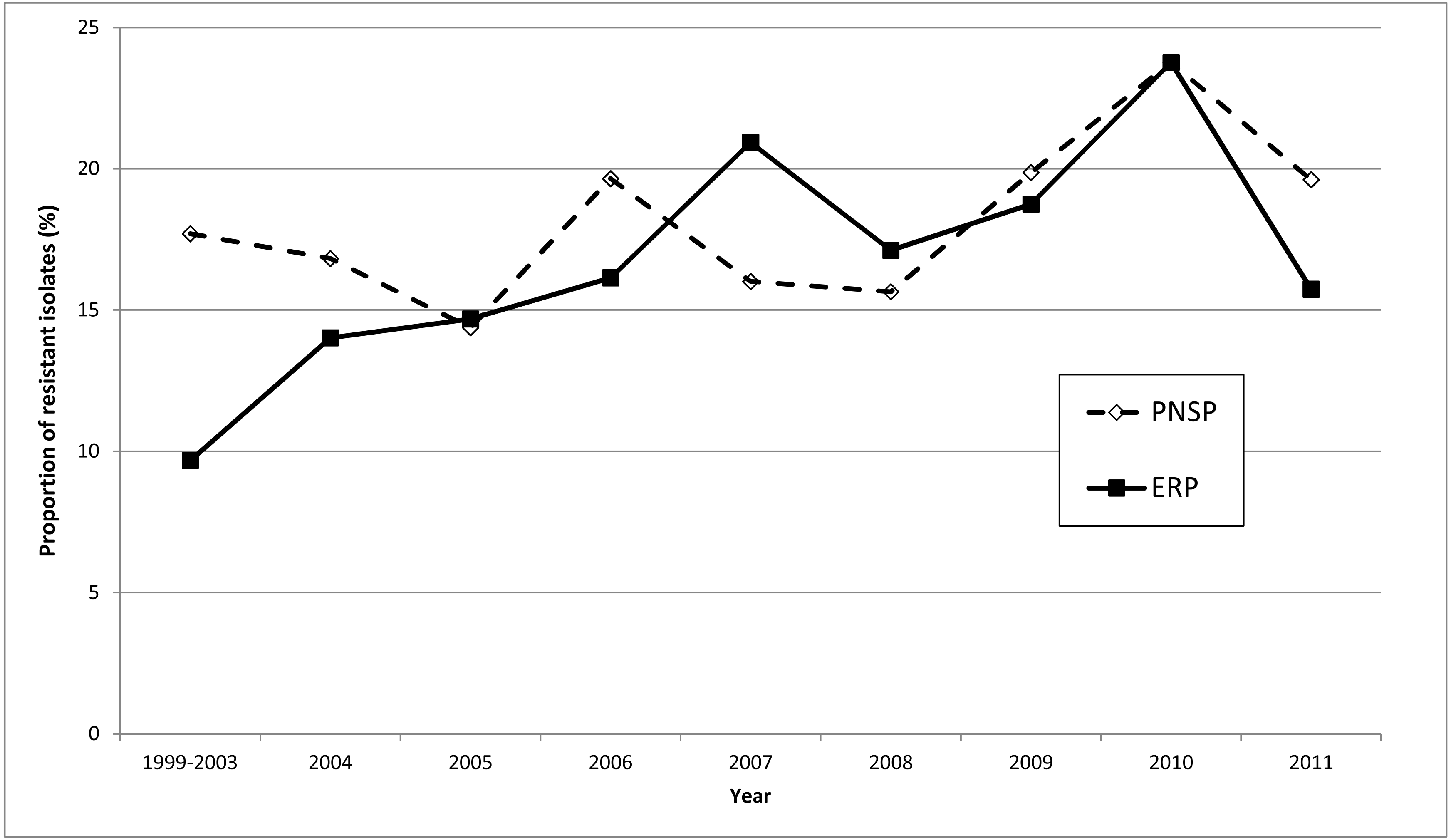

Figure S1. Proportion of penicillin non-susceptible pneumococci (PNSP) and erythromycin resistant pneumococci (ERP) (1999-2011). The period 1999-2003, previously identified as the pre-PCV7 period, was analyzed together.
