# Supplementary material for: The Majority of Adult Pneumococcal Invasive Infections in Portugal Are Still Potentially Vaccine Preventable in Spite of Significant Declines of Serotypes 1 and 5
Source: PLoS One. 2013 Sep 16;8(9):e73704. doi: 10.1371/journal.pone.0073704 (PMC3774749; doi:10.1371/journal.pone.0073704)
Supplement: Table S1 — Number of isolates expressing serotypes included in the 13-valent conjugate vaccine but not included in the 7-valent conjugate vaccine causing invasive infections in Portugal (2008–2011). (PDF) [file pone.0073704.s002.pdf]

Table S1. Number of isolates expressing serotypes included in the 13-valent conjugate vaccine but not included in the 7 and 10-valent conjugate vaccines causing invasive infections in Portugal (2008-2011).

Serotype	No. isolates in each year (%)			
	2008	2009	2010	2011
1	55 (13.4)	48 (10.7)	22 (5.4)	17 (4.1)
3	51 (12.5)	53 (11.8)	59 (14.6)	48 (11.6)
5	12 (2.9)	9 (2.0)	4 (1.0)	0 (0)
6A	6 (1.5)	8 (1.8)	2 (0.5)	1 (0.2)
7F	48 (11.7)	48 (10.7)	35 (8.7)	43 (10.4)
19A	48 (11.7)	33 (7.4)	44 (10.9)	38 (9.2)
