# Supplementary material for: The Majority of Adult Pneumococcal Invasive Infections in Portugal Are Still Potentially Vaccine Preventable in Spite of Significant Declines of Serotypes 1 and 5
Source: PLoS One. 2013 Sep 16;8(9):e73704. doi: 10.1371/journal.pone.0073704 (PMC3774749; doi:10.1371/journal.pone.0073704)
Supplement: Table S2 — Capsular types of the isolates recovered from CSF between 2009 and 2011. (PDF) [file pone.0073704.s003.pdf]

Table S2. Capsular types of the isolates recovered from CSF between 2009 and 2011.

	No. isolates		
Serotype	CSF	non-CSF	OR ($CI_{95\%}$)
3	9	151	0.69 (0.30-1.41)
19A	9	106	1.02 (0.44-2.11)
**19F**	**7**	**19**	**4.69 (1.62-12.05)**
**6B**	**6**	**13**	**5.84 (1.78-16.95)**
11A	5	34	1.81 (0.54-4.81)
**16F**	**5**	**13**	**4.82 (1.32-14.80)**
7F	5	121	0.47 (0.15-1.17)
22F	4	51	0.94 (0.24-2.65)
33A	4	12	4.14 (0.95-14.00)
1	3	84	0.41 (0.08-1.29)
14	3	103	0.33 (0.07-1.02)
10A	3	18	2.04 (0.38-7.17)

[a] Odds ratio (OR) and 95% confidence intervals ($CI_{95\%}$) are shown for serotypes that were represented by at least 3 isolates.

[b] In bold are the serotypes with significant p-values after FDR correction.
